# The Role of Genetic Testing in the Identification of Young Athletes with Inherited Primitive Cardiac Disorders at Risk of Exercise Sudden Death

**DOI:** 10.3389/fcvm.2016.00028

**Published:** 2016-08-26

**Authors:** Francesco Danilo Tiziano, Vincenzo Palmieri, Maurizio Genuardi, Paolo Zeppilli

**Affiliations:** ^1^Istituto di Medicina Genomica, Università Cattolica del Sacro Cuore, Roma, Italy; ^2^Unità di Medicina dello Sport, Fondazione Policlinico “A. Gemelli”, Università Cattolica del Sacro Cuore, Roma, Italy

**Keywords:** athletes, sudden cardiac death, genetics, medical, hypertrophic cardiomyopathy, long QT syndrome, arrhythmogenic right ventricular displasia, isolated non-compact myocardium

## Abstract

Although relatively rare, inherited primitive cardiac disorders (IPCDs) in athletes have a deep social impact since they often present as sudden cardiac death (SCD) of young and otherwise healthy persons. The diagnosis of these conditions is likely underestimated due to the lack of shared clinical criteria and to the existence of several borderline clinical pictures. We will focus on the clinical and molecular diagnosis of the most common IPCDs, namely hypertrophic cardiomyopathies, long QT syndrome, arrhythmogenic right ventricular cardiomyopathy, and left ventricular non-compaction. Collectively, these conditions account for the majority of SCD episodes and/or cardiologic clinical problems in athletes. In addition to the clinical and instrumental tools for the diagnosis of IPCD, the viral technological advances in genetic testing have facilitated the molecular confirmation of these conditions. However, genetic testing presents several issues: the limited sensitivity (globally, around 50%), the low prognostic predictive value, the probability to find pathogenic variants in different genes in the same patient, and the risk of non-interpretable results. In this review, we will analyze the pros and cons of the different clinical approaches for the presymptomatic identification, the diagnosis and management of IPCD athletes, and we will discuss the indications to the genetic testing for patients and their relatives, particularly focusing on the most complex scenarios, such as presymptomatic tests, uncertain results, and unexpected findings.

## Introduction

Inherited primitive cardiac disorders (IPCDs) comprise a wide and heterogeneous group of conditions. Two major subcategories of IPCDs are universally recognized: primitive cardiomyopathies and primitive electric disorders of the heart. Primitive cardiomyopathies can be defined as disorders characterized by morphologically and functionally abnormal myocardium, in the absence of other diseases that can cause the observed cardiac phenotype (1). This definition is aimed at distinguishing primitive cardiomyopathies from conditions in which the cardiac involvement is secondary to a systemic disorder. Primitive electric disorders are conditions characterized by the presence heart electric conduction disturbances with a morphologically normal myocardium (2). However, while the pathophysiological mechanisms are different, the two groups of IPCDs encompass a continuous spectrum of diseases: rhythm disturbances occur in cardiomyopathies secondary to myocardial disarray and are the leading cause of death. Most IPCDs are Mendelian conditions, most commonly transmitted as autosomal dominant traits with incomplete penetrance, show familial recurrence and have a high degree of genetic heterogeneity (many genes causing the same or similar phenotypes).

First level diagnostic tools are standard cardiologic investigations, such as electrocardiography (ECG) and/or echocardiography (EchoCG), which can be supplemented with cardiac magnetic resonance and/or Holter ECG when needed (3). These will not be discussed in this review, since papers more focused on clinical aspects have been recently published (3).

The prevalence of IPCDs in general is likely underestimated, due to the reduced penetrance (see below), and to the existence of a wide gray zone between normal and definitely pathological instrumental findings.

In the setting of sports medicine, the identification and clinical management of IPCD becomes even more complex: the younger age of the population at risk and the presence of common features between IPCD and athlete heart may delay or prevent a timely diagnosis of these conditions; additionally, the extreme exertion may exacerbate underlying cardiac defects. The onset of IPCD symptoms in athletes is often dramatic, since these are the leading cause of sudden cardiac death (SCD). Thus, presymptomatic identification of affected individuals is of paramount relevance. Following an IPCD diagnosis, it is crucial to evaluate the eligibility for competitive or recreational sports (that implies a lesser cardiac impact), to establish the prognosis, to prevent the occurrence of fatal events, and, last but not least, to evaluate the risk for the athlete’s offspring and sibship.

The main relevance of genetic testing for IPCDs is in identifying at risk subjects or to solve diagnostic uncertainties. The most commonly altered genes involved in IPCDs are listed in Table 1. The introduction of next-generation sequencing (NGS) platforms in diagnostic laboratories and the consequential reduction of costs of molecular analysis per patient have improved the diagnostic yield and reduced the time interval between sampling and final reports. However, the data accrued have revealed the complexity of the genetics of IPCD (4–10). With the traditional Sanger sequencing diagnostic approach, single genes were investigated sequentially, one after another, and often testing was interrupted when a pathogenic or likely pathogenic variant was found. NGS-based approaches allow to test several genes simultaneously. As a consequence, the finding of individuals carrying ≥2 rare pathogenic or potentially pathogenic variants in the same or different genes has become not uncommon. At the same time, there has been a surge in the numbers of variants of uncertain significance (VUS) detected. Moreover, it has become clear that allelic variants in the same gene can be associated with different phenotypes, increasing the difficulties inherent to the interpretation of genetic test results. These findings suggest that the variable phenotypic spectrum of IPCDs cannot be only accounted for by classical Mendelian mechanisms, and point toward the involvement of an oligogenic model with strong environmental influences.

**Table 1:** **The genes most commonly altered in cardiomyopathies**.

Gene name	Gene symbol	Frequency (%)
**Hypertrophic cardiomyopathy**		
Beta-myosin heavy chain	*MYH7*	15–25
Cardiac myosin-binding protein C	*MYBPC3*	15–25
Cardiac troponin T	*TNNT2*	<5
Cardiac troponin I	*TNNI3*	<5
Alpha-tropomyosin	*TPM1*	<5
LIM-binding domain 3	*LBD3*	1–5
Ventricular regulatory myosin light chain	*MYL2*	<2
Myosin light chain 3	*MYL3*	<1
Cardiac muscle alpha actin	*ACTC1*	<1
**Long QT syndrome**		
Potassium channel, voltage gated, member 1 (Kv7.1; LQT1)	*KCNQ1*	40–55
Potassium channel, voltage gated, member 2 (Kv11.1; LQT2)	*KCNH2*	30–45
Sodium channel, voltage gated, type V, α subunit (Nav1.5; LQT3)	*SCN5A*	5–10
**Arrhythmogenic right ventricular cardiomyopathy**		
Plakophilin 2	*PKP2*	10–45
Desmoplakin	*DSP*	10–15
Desmoglein	*DSG2*	7–10
Desmocollin 2	*DSC-2*	2
Junction plakoglobin	*JUP*	<1

*Genes that are rarely mutated have not been included [adapted from Bos et al. (11), Mizusawa et al. (12), and Iyer and Chin (13)]*.

In this review, we will focus on the clinical and molecular diagnosis of the most common IPCDs in athletes, namely hypertrophic cardiomyopathies (HCMs), long QT syndrome (LQTS), arrhythmogenic right ventricular cardiomyopathy (ARVC), and left ventricular non-compaction (LVNC). We will also discuss about the *TTN* gene, one of the largest genes in our genome encoding for the giant protein Titin, which is often altered in different clinical conditions. We will finally discuss the prognostic utility of genetic testing, and the counseling approaches to IPCD patients and their families.

## Hypertrophic Cardiomyopathies

Hypertrophic cardiomyopathies belong to the wider spectrum of cardiomyopathies that include also the dilative and restrictive phenotypes. HCM is diagnosed on the basis of left ventricular hypertrophy, in the absence of abnormal loading conditions. The estimated prevalence in the young adults is about 0.1–0.2%, which does not likely reflect the prevalence in the general population that is expected to be higher (14): available data have been obtained through clinical studies, and thus do not take into account the ascertainment biases related to later onset of symptoms and to the presence of borderline patients (14). According to studies performed in the US, HCM is the most common cause of SCD in young athletes (15–17); it is noteworthy that in countries where the preparticipation screening by ECG is mandatory by law, the incidence of HCM as cause of SCD among athletes is dramatically lower (18).

About half of HCM cases are familial with an autosomal dominant pattern of inheritance. More than 20 genes have been related to HCM: b-myosin heavy chain (*MYH7*) and cardiac myosin-binding protein C (*MYBPC3*) account for about 50% of the cases; the other genes are rarely affected, with some involved in a single family so far. Incomplete penetrance is an important issue for HCM management. Environmental factors (such as intense training) and/or modifier genes may increase the risk of clinical manifestations especially during exercise or sport. If this was the case, one should expect to find more frequently clinical/instrumental signs of HCM among athletes compared with the general population. However, to the best of our knowledge, there are no available data on the true prevalence of HCM among professional athletes. Since the main complication of this condition is sudden death, the development of primary prevention programs aimed at identifying at risk subjects is very important, despite the relatively low frequency of HCM (16–18). There are not yet enough sensitive clinical markers that may help to identify HCM patients at risk of sudden death. The most reliable predictors are family history of sudden death related to HCM, syncope or presyncope events, ventricular tachyarrhythmia, marked hypotension during training, extreme left ventricle hypertrophy, and extended late enhancement at cardiac MRI (19–22), but their performance is far from satisfactory. A quantitative approach for the assessment of the risk of sudden death in HCM has been reported by O’Mahony et al. (23), the so-called HCM risk SCD. In this case, the risk is estimated on the basis of data collected in a retrospective longitudinal study by taking into account different variables.

Rather than for patients with clear HCM phenotypes, genetic testing may be useful for the proper interpretation of borderline patients falling in the gray zone. However, reduced penetrance, genetic heterogeneity, and high VUS frequency make the interpretation of the clinical significance of genetic variants challenging. Furthermore, a preliminary NGS-based study reported the occurrence of double heterozygosity in a high proportion of HCM patients, 2 of the 11 patients with pathogenic variants. These patients were reported as having a more severe phenotype compared with patients with a single disease causing variant (24).

With regard to practical implications for molecular diagnosis, according to the guidelines of the European Society of Cardiology (ESC), genetic testing could be offered to all patients fulfilling the HCM diagnostic criteria. Irrespective of the sequencing methodology employed, genetic analysis should include the most commonly implicated sarcomere protein genes (1). Following the identification of a definite pathogenic variant in the proband, genetic testing can be offered to all relatives on a voluntary basis. If no causative variants are found in the proband, relatives should be advised to undergo clinical reassessment should symptoms of HCM manifest.

## Long QT Syndrome

Long QT syndrome is defined by the finding of a prolonged QT interval in standard ECG recording. It is generally accepted that the normal duration of the QT interval is 0.37–0.44 s. Based on this criterion, the diagnosis of LQTS is apparently easy, but 15% of subjects in the general population have a QT interval >0.44 s (0.44–0.47 s) and 25–35% of individuals with a pathogenic variant in one of the LQTS genes has a normal QT interval (25, 26). This latter observation deserves some additional comments: at this stage, it is very difficult to establish whether the finding of a variant considered disease causing in asymptomatic patients is due to reduced penetrance or if, in the light of more recent concepts of molecular genetics, it is the consequence of a wrong interpretation, and the observed DNA change in a VUS, or even a rare benign, not clinically relevant, variation.

Since the diagnostic value of QT interval measurement on its own is not sufficient, a scoring method based on multiple parameters is currently used [Table 2; (27)]. LQTS belongs to the wider nosologic group of the channelopathies, and its cumulative prevalence is about 1/2,500: as in the case of HCM, this is likely an underestimate, due to the wide phenotypic heterogeneity. The majority of cases are familial (about 90%). As in the case of HCM, few genes account for the vast majority of cases: specifically, defects in *KCNQ1, KCNH2*, and *SCN5A* are found in about 80% of patients. Double heterozygotes are not uncommon: two pathogenic or likely pathogenic variants in different genes are observed in about 10% of patients, and these often display more severe phenotypes (28).

**Table 2:** **LQTS diagnostic criteria [from Schwartz and Crotti (25)]**.

	Points
**Electrocardiographic findings^a^**	
A. QTc^b^	
≥480 ms	3
460–479 ms	2
450–459 ms (in males)	1
B. QTc^b^ fourth minute of recovery from exercise stress	
Test ≥480 ms	1
C. Torsade de pointes^c^	2
D. T wave alternans	1
E. Notched T wave in 3 leads	1
F. Low heart rate for age^d^	0.5
**Clinical history**	
A. Syncope^c^	
With stress	2
Without stress	1
B. Congenital deafness	0.5
**Family history**	
A. Family members with definite LQTS^e^	1
B. Unexplained sudden cardiac death below 30 years of age among immediate family members^e^	0.5

*^a^In the absence of medications or disorders known to affect these electrocardiographic features*.

*^b^QTc calculated by Bazett’s formula where QTc = QT/√RR*.

*^c^Mutually exclusive*.

*^d^Resting heart rate below the second percentile for age*.

*^e^The same family member cannot be counted in A and B*.

*Score: ≤1 point: low probability of LQTS; 1.5–3 points: intermediate probability of LQTS; ≥3.5 points high probability*.

The diagnosis of LQTS in athletes is complicated by the correlation between duration of the QTc interval and exercise, and the extreme variation of heart rate reached by athletes. In two studies, the prevalence of LQT was 0.6 and 0.4% in an Italian and a British athlete population, respectively (29, 30), that is about 10- to 15-fold higher than in the general population. In the British study, molecular screening of *KCNQ1, KCNH2*, and *SCN5A* was performed in five of the seven patients with LQT, three of whom had a QT interval >0.50 s and additional signs reinforcing the suspicion of LQTS; a pathogenic variant was found only in one patient. However, the apparently low yield of genetic testing in this cohort of patients could be related to technical limitations. A proper diagnosis of LQTS in athletes is of particular relevance: besides the obvious implications for the patient and the family, it entails also important career implications, since it is suggested that it may represent a contraindication to competitive sport disciplines involving moderate- and high-intensity strenuous exertion (31–33).

## Arrhythmogenic Right Ventricular Cardiomyopathy

Arrhythmogenic right ventricular cardiomyopathy is a cardiac muscle disease characterized by life-threatening ventricular arrhythmias. The estimated prevalence is about 1:2,500–5,000. ARVC is considered one of the major causes of sudden death in young individuals and in athletes (18). ARVC is generally associated with ECG alterations, including negative T wave in right precordial leads, ventricular arrhythmias with a left bundle branch block morphology, epsilon waves, and others. However, some of these abnormalities are not specific and may be found in other pathological conditions with a different prognosis, such as myocarditis (34). The extensive use of ECG screening may help the sports physician to suspect the diagnosis, while genetic testing may be very useful for the differential diagnosis with more benign conditions.

Cardiac pathology shows dystrophy of the right ventricular myocardium with fibrofatty replacement. The clinical picture may include a subclinical asymptomatic phase; ventricular fibrillation, or an electrical disorder with palpitations and syncope, due to tachyarrhythmias of right ventricular origin, may be the first presentation. Most ARVC genes encode for proteins of mechanical cell junctions (*DSC2, DSG2, DSP, PKP2, JUP*, and *DES*), while others encode for structural proteins of the nuclear membrane (*LMNA* and *TMEM43*) or membrane channels (*RYR2*). ARVC has an autosomal dominant pattern of inheritance with incomplete penetrance. Pathogenic variations in the nine ARVC genes identified so far account for about 50% of cases. Double heterozygotes have been reported also in this condition. Clinical diagnosis may be achieved by demonstrating functional and structural alterations of the right ventricle, depolarization and repolarization abnormalities, arrhythmias with left bundle branch block morphology, and fibrofatty replacement upon endomyocardial biopsy [see Basso et al. (35) for a review]. Albeit rare, the diagnosis of ARVC is of crucial importance for athletes, due to the risk of sudden death: the condition was originally described as the most common cause of death in sportsmen. However, it is now evident that the condition has a wide phenotypic variability, including very mild asymptomatic cases: sport activity may increase the risk of ventricular arrhythmias in asymptomatic subjects with pathogenic variants in desmosomal genes (36).

## Left Ventricular Non-Compaction

Left ventricular non-compaction is due to the precocious arrest of myocardial compaction during the first weeks of the embryonic development. This causes persistence of prominent trabeculae in the ventricular cavity. The disease spectrum is very wide: the first reported cases were of patients with a marked dilation of the left ventricle and high risk of death (37–40), but asymptomatic and barely progressive segmental forms have also been reported, in which the lack of compaction involves only part of the left ventricle. LVNC is a rare disorder with prevalence <0.1%, although it has been increasingly diagnosed over the last few years. Similar to many other rare disorders, with increasing knowledge its diagnosis has become more common, and among newly identified cases, there is an increasing proportion of asymptomatic subjects, including athletes, with mild phenotypic expression. Indeed, heart hypertrabeculation has been observed in up to 18.3% of athletes (41), about 8% of which fulfill the diagnostic criteria of LVNC. In our experience, a multiparametric evaluation, based on morphological and functional parameters, such as the thickness of the residual compact layer and the presence of major conduction defects and arrhythmias, may help to discriminate between true cardiomyopathies and “benign” forms of LVNC (42). In the latter group of patients, the risk of sudden death, heart failure, and life-threatening arrhythmias is likely low. In any case, close follow-up is still recommended.

Genetic testing is not particularly useful for the molecular confirmation of LVNC for at least two reasons: the detection rate of pathogenic variants is relatively low, about 40%, and the genes involved in LVNC are also responsible also for other cardiomyopathies, complicating the interpretation of positive test results (43). Based on these findings, it has been proposed that LVNC may be a phenotypic variant of other cardiomyopathies, characterized by impaired general development of the sarcomeric proteins: this pathogenic model is supported by the cooccurrence in the same family of LVNC and different cardiomyopathies. It is conceivable that genetic background and environmental factors may play a relevant role in the onset of LVNC (44).

Regarding the relationship with sport activity, the number of incidental diagnoses has increased over time, often in asymptomatic athletes. Of note, hypertrabeculation of the left ventricle may physiologically occur in athletes, particularly in elite and black sportsmen. Thus, it is crucial to distinguish between the true cardiomyopathy and the benign segmental LVNC for the assessment of the risk of serious life-threatening events (45).

## Titin: A Titan or A Giant with Clay Feet?

Although if it is only one of the genes involved in cardiomyopathies, the titin gene (*TTN*) deserves a separate discussion due to its peculiarities. *TTN* is one of the largest genes in our genome and encodes for the largest human protein. The titin protein has several functions in both cardiac and skeletal muscle. Due to the size, prior to the advent of NGS, the mutational analysis of *TTN* was limited to few exons. The exact number of isoforms is unknown, although it has been estimated that at least one-third of *TTN* exons may give raise to alternative splicing events (46, 47). *TTN* has been associated with both dominant and recessive disorders and is currently considered one of the most commonly altered genes in human disease (48), causing at least 10 different conditions, involving skeletal muscle, heart, or both. However, accruing data on genomic variations in the general population have shown that rare *TTN* variants overall are common, with at least 2–3% of healthy individuals bearing monoallelic truncating mutations. Rare and private missense variants are extremely common as well (47, 49). We should then expect a prevalence of recessive pathogenic variants of at least 1/4,000–10,000, much higher than the cumulative prevalence of titinopathies. Thus, it seems that at least a part, if not the majority, of truncating *TTN* variants is benign and does not cause pathological phenotypes on their own. The lack of pathogenicity of *TTN* alleles potentially causing complete loss of function could be explained by alternative splicing events rescuing the gene function.

Based on these observations, the pathogenic role of *TTN* variants should be assessed cautiously, especially for the potential application to presymptomatic-predictive testing in healthy relatives of patients in whom *TTN* alterations have been detected. It is likely that in the few next years, with accruing genomic data in the general population and the spreading of NGS platforms for the diagnosis of cardiomyopathies, the pathogenicity of *TTN* variants will be largely elucidated.

## Discussion

In this review, we have highlighted critical aspects associated with the clinical and genetic diagnosis of the IPCDs. The most recent findings on the variability of the human genome are quickly changing the approach to DNA variant interpretation. Indeed, a systematic assessment of variants, including those previously interpreted as pathogenic, is ongoing for several genes associated with inherited conditions. Overall, the following issues are associated with all types of IPCD and complicate their diagnosis and management: (1) wide genetic heterogeneity, (2) incomplete penetrance, (3) relatively high frequency of double heterozygotes, and (4) effect of environmental factors (largely unknown as well, besides sport activity). In the light of these characteristics, IPCDs could be considered as complex traits determined by the predominant effect of single gene variants, rather than as monogenic disorders. Considering IPCDs as oligogenic multifactorial disorders has three main implications: (1) genotype–phenotype correlations are unclear, (2) difficulty in establishing prognosis and risks for patients, and consequently, (3) genetic testing has a limited predictive power both in affected patients and in asymptomatic relatives at risk. Therefore, in our opinion, the use of the risk figures estimated according to Mendelian inheritance is not fully appropriate for predictive purposes. It could be useful to develop a risk assessment model similar to those applied for the familial predisposition to breast cancer, taking into account the presence of multiple factors (e.g., family history, level of exposure to physical activity, presence of multiple gene variants) (50). Indeed, with few exceptions (51–53), due to poor genotype–phenotype correlations, results of clinical investigations provide better prognostic information than those of genetic testing.

These problems become even more complicated in athletes, who can display some features resembling those of IPCD as a consequence of physiological rearrangements of the myocardium with training. These subjects, who are mostly young, present some additional issues: the ascertainment of a variant known to be the cause of an IPCD phenotype may have strong implications for the prosecution of their sport career, for reproductive choices, and for their families. The offer of a predictive test for relatives should be considered with caution, and only when the pathogenicity of the variant detected in the proband has been clearly established according to consensus criteria (54). The opportunity of testing underage relatives of athletes should also be carefully scrutinized, especially when presymptomatic diagnosis may be beneficial, such as in the case of LQTS, which can manifest as infant sudden death.

Variant interpretation is the main issue in molecular diagnosis of IPCDs, and it is further exacerbated by the small size of many pedigrees. This hampers analysis of variant segregation with respect to the phenotype, one of the most useful points of evidence for clinical interpretation of genetic variants, as well the estimation of penetrance values. Ideally, novel or unclassified variants should be validated by functional studies; however, with few exceptions, these are not performed in clinical diagnostic laboratories and are associated with several issues, such as the choice of the cellular model and feasibility, since most sarcomeric mRNAs are quite large and not easily manageable. In conclusion, unless validated functional tests have been performed, the main hints suggesting pathogenicity of a variant are its identification in different patients or *de novo* occurrence.

Another open issue is the significance of double heterozygosity and related counseling. So far, it is common opinion that these subjects may in general display a more severe phenotype but the cohorts published so far are too small to draw definite conclusions.

*TTN* deserves separate considerations. In particular, given the difficulties inherent with *TTN* molecular testing and interpretation, one might wonder whether it is appropriate to include this gene in diagnostic panels and in genetic reports for patients, or rather, whether it should still be investigated in research settings for epidemiological purposes. These might shed light on the pathogenicity of *TTN* truncating and missense variants, as well as and on their clinical relevance; indeed, it remains to be established if *TTN* variants act as main phenotypic drivers and/or as a risk factor for the appearance of the clinical manifestation of some IPCDs, insufficient alone to determine a phenotype.

Similar to *TTN*, also in HCM the large amount of whole exome data that are accumulating in the different databases is disclosing that presumptive pathogenic variants can be found in “controls” at a higher rate than expected for the prevalence of the condition (55–57). On the one hand, this excess of pathogenic variants could be accounted for by reduced penetrance: carriers identified in the general population may or may not develop signs of HCM over time, but should be considered as asymptomatic subjects. On the other hand, these findings may indicate that the effects of these gene variants are too weak to cause appearance of the phenotype on their own. In conclusion, the refinement of clinical diagnosis of IPCD, coupled with the new technological tools available in molecular genetics, has opened the Pandora box of cardiac primitive defects. Now, the pieces of this puzzle need to be reconstructed in order to provide patients and athletes with more accurate information and best care. But, there is still a long way to go.

## Author Contributions

All authors listed, have made substantial, direct and intellectual contribution to the work, and approved it for publication.

## Conflict of Interest Statement

The authors declare that the research was conducted in the absence of any commercial or financial relationships that could be construed as a potential conflict of interest.
